# Association Between Pathogens Detected Using Quantitative Polymerase Chain Reaction With Airway Inflammation in COPD at Stable State and Exacerbations

**DOI:** 10.1378/chest.14-0764

**Published:** 2014-08-07

**Authors:** Bethan L. Barker, Koirobi Haldar, Hemu Patel, Ian D. Pavord, Michael R. Barer, Christopher E. Brightling, Mona Bafadhel

**Affiliations:** From the Institute for Lung Health (Drs Barker, Barer, and Brightling), National Institute for Health Research Respiratory Biomedical Research Unit, and the Department of Infection, Immunity, and Inflammation (Drs Barker, Barer, and Brightling and Ms Haldar), University of Leicester, Leicester; the Department of Clinical Microbiology (Ms Patel and Dr Barer), University Hospitals of Leicester National Health Service Trust, Leicester; and the Respiratory Medicine Unit (Drs Pavord and Bafadhel), Nuffield Department of Clinical Medicine, University of Oxford, Old Road Campus, Oxford, England.

## Abstract

**BACKGROUND:**

Relationships between airway inflammation and respiratory potentially pathogenic microorganisms (PPMs) quantified using quantitative polymerase chain reaction (qPCR) in subjects with COPD are unclear. Our aim was to evaluate mediators of airway inflammation and their association with PPMs in subjects with COPD at stable state and during exacerbations.

**METHODS:**

Sputum from 120 stable subjects with COPD was analyzed for bacteriology (colony-forming units; total 16S; and qPCR targeting *Haemophilus influenzae*, *Moraxella catarrhalis*, and *Streptococcus pneumoniae*), differential cell counts, and inflammatory mediators using the Meso-Scale Discovery Platform. Subjects were classified as colonized if any PPM was identified above the threshold of detection by qPCR. Symptoms were quantified using the visual analog scale.

**RESULTS:**

At stable state, 60% of subjects were qPCR positive for *H influenzae*, 48% for *M catarrhalis*, and 28% for *S pneumoniae*. Elevated sputum concentrations of IL-1β, IL-10, and tumor necrosis factor (TNF)-α were detected in samples qPCR positive for either *H influenzae* or *M catarrhalis*. Bacterial loads of *H influenzae* positively correlated with IL-1β, IL-8, IL-10, TNF-α, and symptoms; and *M catarrhalis* correlated with IL-10 and TNF-α. *H influenzae* qPCR bacterial load was an independent predictor of sputum TNF-α and IL-1β. In 55 subjects with paired exacerbation data, qPCR bacterial load fold change at exacerbation in *M catarrhalis* but not *H influenzae* correlated to changes in sputum TNF-α and IL-1β concentrations.

**CONCLUSIONS:**

At stable state, *H influenzae* is associated with increased airway inflammation in COPD. The relationship between bacterial load changes of specific pathogens and airway inflammation at exacerbation and recovery warrants further investigation.

COPD is characterized by irreversible airflow obstruction and airway inflammation. The disease course is punctuated by exacerbation episodes,^1^ which are often associated with increased airway inflammation,^2^ viruses, and bacteria.^3^ Bacteria are isolated from sputum cultures in 30% to 40% of subjects with stable COPD^3‐7^ and found in approximately 50% of subjects during exacerbation episodes.^7,8^ Colonization with bacteria is associated with worsened health status,^6^ reduced lung function,^9^ and an increase in the frequency and severity of exacerbations.^10^ Additionally, patients with positive sputum cultures have an associated increased inflammatory response detected by elevated levels of sputum neutrophils,^11^ IL-8,^12‐14^ IL-6,^13^ tumor necrosis factor (TNF)-α,^13^ myeloperoxidase,^14^ and leukotriene B4.^6^ Both culture-based and culture-independent molecular techniques have shown that *Haemophilus influenzae* is the commonest sputum pathogen in stable COPD.^15,16^ Although studies using culture-based techniques have suggested that airway inflammation is higher in those colonized with *H influenzae*,^15‐17^ relationships between pathogens quantified using molecular techniques and airway inflammation are unclear. In this study using quantitative polymerase chain reaction (qPCR) to measure pathogen-specific bacterial loads, we hypothesized that detection of the respiratory potentially pathogenic microorganisms (PPMs) (*H influenzae, Moraxella catarrhalis*, *Streptococcus pneumoniae*, and *Staphylococcus aureus*) in subjects with COPD is associated with increased airway inflammation at stable state and during exacerbations.

## Materials and Methods

### Subjects

Sputum samples from subjects aged ≥ 40 years and with postbronchodilator FEV_1/_FVC < 0.7 enrolled within an observational COPD exacerbation cohort study were analyzed. The study design and inclusion and exclusion criteria have been described previously.^3^ Subjects with COPD on prophylactic antibiotic therapy were excluded. The study was conducted in accordance with the amended Declaration of Helsinki and was approved by the Leicestershire, Northamptonshire, and Rutland ethics committee (07/H0406/157). All patients gave informed written consent.

### Measurements

Baseline demographic information, smoking history, medication history, and patient-reported history of exacerbations were collected. Subjects were reviewed when clinically stable and during exacerbation episodes; stable visits took place a minimum of 8 weeks after exacerbation episodes. Exacerbations were defined according to the criteria of Anthonisen et al^18^ and health-care use^19^ and treated according to guidelines.^20^ Exacerbation testing and sampling was only performed in subjects who were treatment naive for the episode. At all visits, spirometry and symptom assessment using the St. George’s Respiratory Questionnaire (SGRQ),^21^ the Chronic Respiratory Questionnaire,^22^ and the visual analog scale (VAS)^23^ were undertaken. Spontaneous or induced sputum sampling was collected for analysis of microbiology, differential cell counts, and cytokine analysis, as described later. No differences in inflammatory counts between spontaneous or induced sputum samples were identified in this study, in keeping with other studies,^24,25^ and > 95% of subjects provided spontaneous sputum samples. CT imaging, to investigate bronchiectasis, was not performed as part of the study protocol; however, CT scans performed as part of a routine clinical investigation were interrogated in subjects who entered the study.

### Sputum Assessments

Bacterial load was measured by colony-forming units (CFU) as per standard methods^26^ and quantitative polymerase chain reaction (qPCR) as previously described.^3^ The CFU is a semiquantitative analysis of total live bacterial counts, and a quantitative analysis of both live and dead bacteria was quantified using qPCR, estimating both the total bacterial load based on the abundance of 16S ribosomal subunit encoding genes (total 16S). Pathogen-specific bacterial 16S abundance using qPCR was measured for *H influenzae*, *M catarrhalis*, *S pneumoniae*, and *S aureus* (*Pseudomonas aeruginosa* was not measured by qPCR in this study). Quantification of the total bacterial load of *H influenzae* and *S aureus* was performed using the SYBR Green assay (Life Technologies). The TaqMan assay (Life Technologies) was used to quantify *M catarrhalis* and *S pneumoniae* (target genes and primers listed in e-Table 1). *S aureus* was infrequently detected, and therefore any results relating to this pathogen were not analyzed further. The threshold of detection for pathogen-specific bacterial 16S qPCR analysis and CFU counts was taken as 1 × 10^4^ genome copies/mL and 1 × 10^5^ colonies/mL of sputum, respectively, reflecting previous cutoff thresholds used in this field.^27,28^ Subjects were categorized as pathogen-specific bacterial 16S qPCR positive detection if any qPCR PPM (defined in this study as identification of *H influenzae*, *M catarrhalis*, *S pneumoniae*, and *S aureus*) was identified above the threshold. Subjects were classified as codetection if more than one pathogen-specific bacterial 16S qPCR PPM was identified above the threshold of identification. Sputum was simultaneously processed to obtain cytospins for differential cell counts and cell-free supernatants as previously described.^29^ The sputum inflammatory mediators IL-1β, IL-5, IL-6, IL-8, IL-10, TNF-α, TNFRI, CXCL10, CCL2, CCL3, CCL4, CCL5, CCL13, and CCL17 were measured using the Meso Scale Discovery Platform (Meso Scale Diagnostics, LLC) from sputum supernatants.^25^ All mediators, except IL-10, measured using the MSD were detectable and above the limit of detection in > 75% of samples (IL-10 was above the limit of detection in > 50% of samples; limits of detection presented in e-Table 2).

### Statistical Analysis

Statistical analysis was performed using SPSS version 20 (IBM) and PRISM version 6 (GraphPad Software, Inc). Parametric data were expressed as mean (SEM), nonparametric data as median (interquartile range), and log-normally distributed data as geometric mean (95% CI). Unpaired parametric and nonparametric groups were compared using the Student *t* test and Mann-Whitney test, respectively. The paired *t* test was used to compare matched stable and exacerbation measures of airway bacterial load and sputum inflammatory mediators. For comparison of three or more groups, the one-way analysis of variance was used, with repeated analysis of variance for paired data. The χ^2^ test was used to compare proportions between groups. Pearson correlation coefficient was used to assess correlations between qPCR-measured airway bacterial load and sputum inflammatory mediators. Multivariate stepwise regression analysis was performed to model the effects of bacterial load on proinflammatory cytokine expression, namely sputum TNF-α and IL-1β. Variables that demonstrated significance at the *P* < .10 level using univariate analysis were entered into the model: *H influenzae* and *M catarrhalis* bacterial load, sputum total cell count, percentage sputum neutrophils, and CFU. Exacerbation frequency and % FEV_1_ predicted were also entered into the model for clinical relevance. The regression model did not show violation of multicollinearity or homoscedasticity, and the residuals observed normality. No corrections for multiple mediator measurements were performed. A *P* value < .05 was taken as the threshold of significance for all statistical testing.

## Results

**Figure 1 –:**
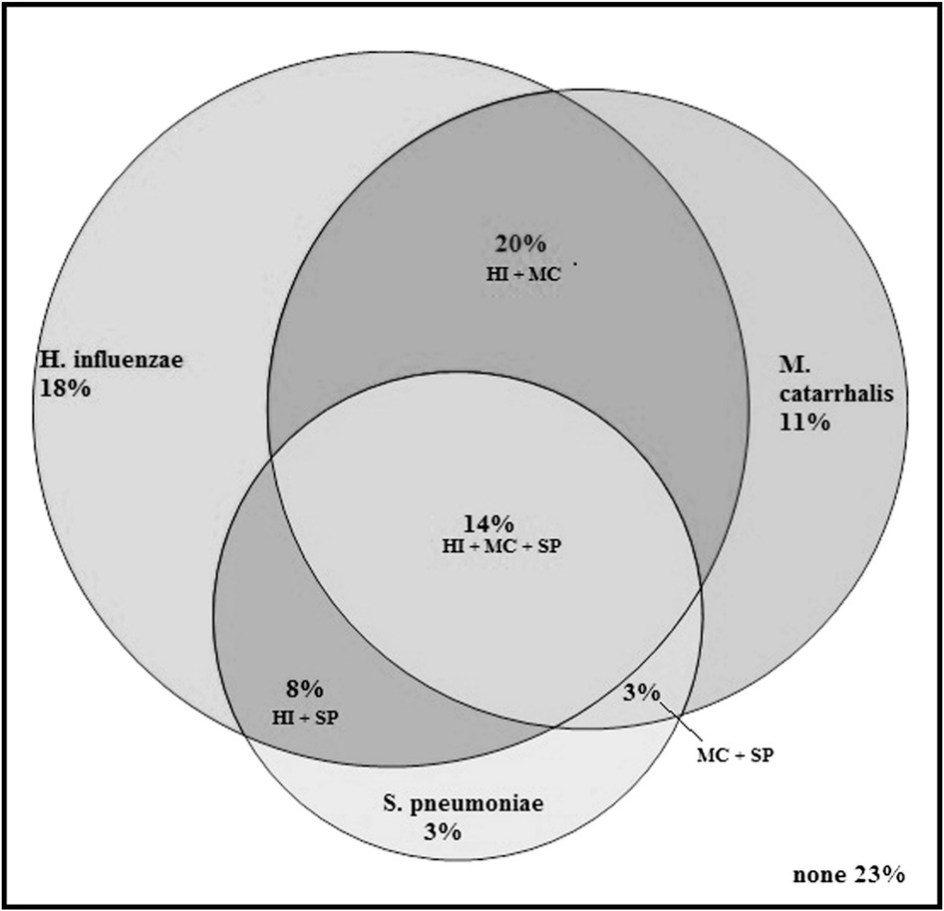
*Proportional Venn diagram depicting the distribution of qPCR codetection at baseline for all subjects. H. influenzae = *Haemophilus influenzae; *HI = *Haemophilus influenzae; *M. catarrhalis = *Moraxella catarrhalis; *MC = *Moraxella catarrhalis; *qPCR = quantitative polymerase chain reaction; S. pneumoniae* = Streptococcus pneumoniae; SP = Streptococcus pneumoniae.

Stable sputum samples with full complement of inflammatory mediators were obtained from 120 subjects (83 men). The clinical characteristics are presented in Table 1. A CT scan was available in 93 subjects (77.5%), and bronchiectasis was detected in 18 (19.4%). There were no significant differences in the clinical parameters between the subjects with COPD with or without detectable pathogen-specific bacterial 16S qPCR PPM. Subjects with PPMs on qPCR had more severe airflow obstruction and increased CFU but not total 16S (Table 1). There was no correlation between total 16S qPCR and inhaled corticosteroid dose, smoking pack-years, or exacerbation frequency. The distribution of qPCR pathogen codetection is presented in Figure 1.

**TABLE 1 ]:** Clinical Characteristics of Subjects According to Whether There Was a Pathogen-Specific Bacterial 16S qPCR PPM Detected Above the Limit of Detection^a^

Characteristic	qPCR PPM Negative (n = 28)	qPCR PPM Positive (n = 92)	*P* Value
Age, mean (range), y	70 (48-87)	69 (43-88)	.37
Ex-smokers, No. (%)	20 (71)	63 (68)	.82
Proportion on ICS, No. (%)	24 (86)	81 (88)	.75
ICS dose, median (IQR), mg (BDP equivalent)	1,300 (800-2,000)	2,000 (800-2,000)	.55
Pack-y history	45 (10-120)	49 (10-153)	.56
FEV_1_, L	1.47 (0.09)	1.32 (0.06)	.20
FEV_1_/FVC %	61 (4)	53 (2)	.05
FEV_1_ %	59 (4)	51 (2)	.06
Exacerbations in previous y	3.8 (0.5)	3.5 (0.3)	.59
SGRQ symptoms, units	62.2 (94.1)	57.2 (2.4)	.31
SGRQ activities, units	70.2 (4.0)	65.7 (2.3)	.35
SGRQ impacts, units	34.7 (3.6)	37.0 (2.0)	.58
SGRQ total, units	49.6 (3.3)	48.7 (1.9)	.82
CRQ emotion, units	4.7 (0.2)	4.7 (0.2)	.97
CRQ fatigue, units	3.6 (0.2)	3.6 (0.1)	.92
CRQ dyspnea, units	3.4 (0.2)	3.3 (0.1)	.69
CRQ mastery, units	4.9 (0.3)	4.9 (0.2)	.82
CRQ total, units	4.2 (0.2)	4.1 (0.1)	.10
VAS cough, mm	35 (6)	39 (3)	.45
VAS dyspnea, mm	50 (5)	47 (3)	.64
VAS sputum production, mm	29 (5)	38 (3)	.13
VAS sputum purulence, mm	33 (5)	29 (3)	.55
Log CFU/mL, mean (95% CI)	6.6 (5.4-7.8)	7.5 (5.9-9.1)	< .01
Log 16S genome copies/mL, mean (95% CI)	8.2 (6.8-9.4)	8.4 (6.5-9.8)	.37
Sputum total cell count, × 10^6^/g	2.5 (1.8-3.7)	3.6 (2.5-5.1)	.27
Sputum neutrophils, mean (range), %	64 (4)	72 (2)	.09
Sputum neutrophil count, × 10^6^/g (geometric mean, 95% CI)	1.6 (0.9-2.8)	2.4 (1.7-3.4)	.22
Sputum eosinophils, % (geometric mean, 95% CI)	1.6 (1.1-2.2)	1.3 (0.9-1.9)	.61
Proportion with bronchiectasis, No. (%)^b^	4 of 19 (21)	14 of 74 (18)	.74

Data expressed as mean (SEM) unless otherwise stated. BDP = beclomethasone dipropionate; CFU = colony-forming units; CRQ = Chronic Respiratory Questionnaire; ICS = inhaled corticosteroid; PPM = potentially pathogenic microorganism; qPCR = quantitative polymerase chain reaction; SGRQ = St. George’s Respiratory Questionnaire; VAS = visual analog scale.

a > 1 × 10^4^ genome copies/mL.

b CT scans available in 93 out of 120 subjects.

### qPCR and Inflammation During Stable State

**Figure 2 –:**
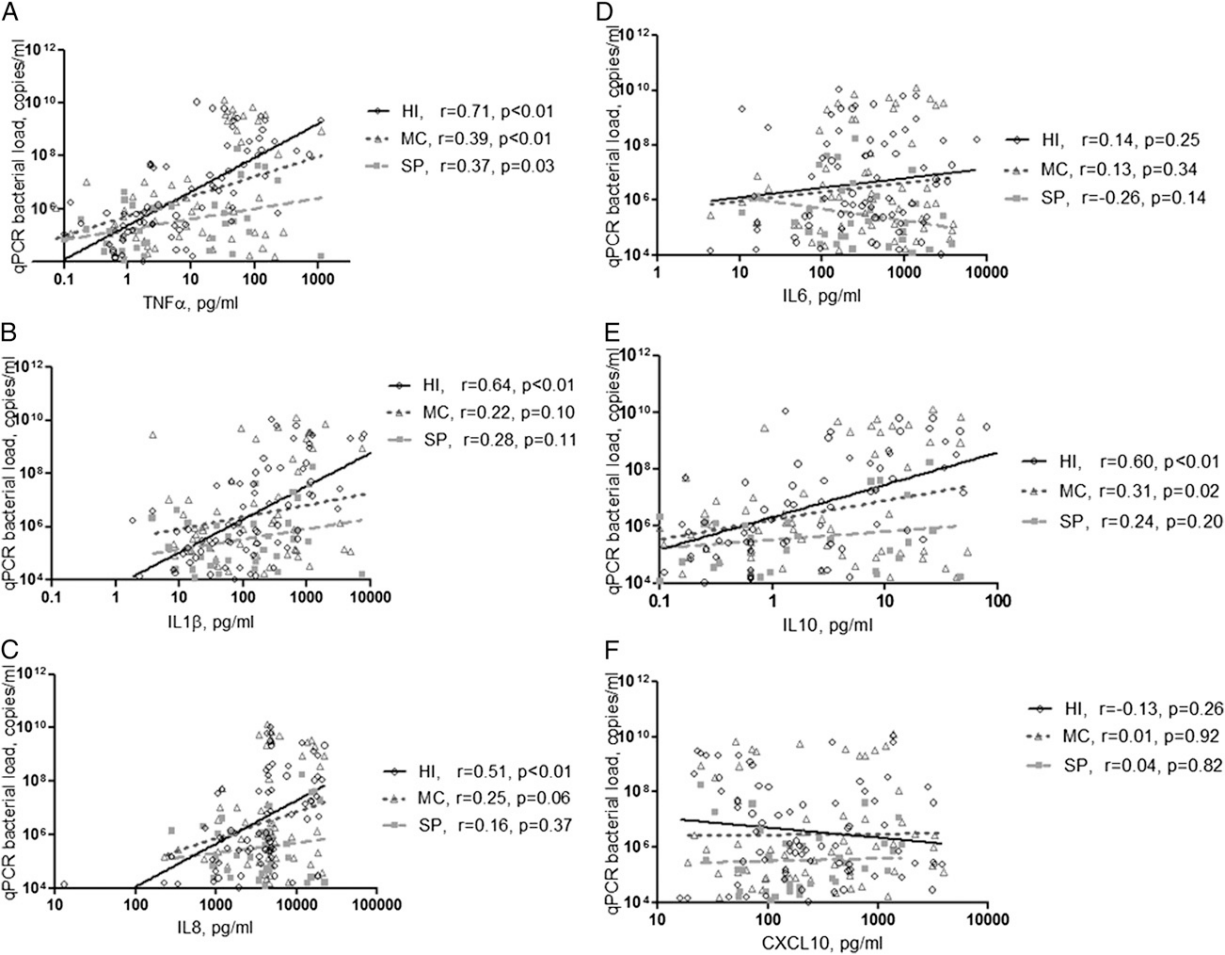
*A-F, Correlations between the sputum mediator concentrations (A) TNF*α*, (B) IL1*β*, (C) IL8, (D) IL6, (E) IL10, and (F) CXCL10 and sputum bacterial load of HI, MC, and SP. TNF = tumor necrosis factor. See Figure 1 legend for expansion of other abbreviations.*

**Figure 3 –:**
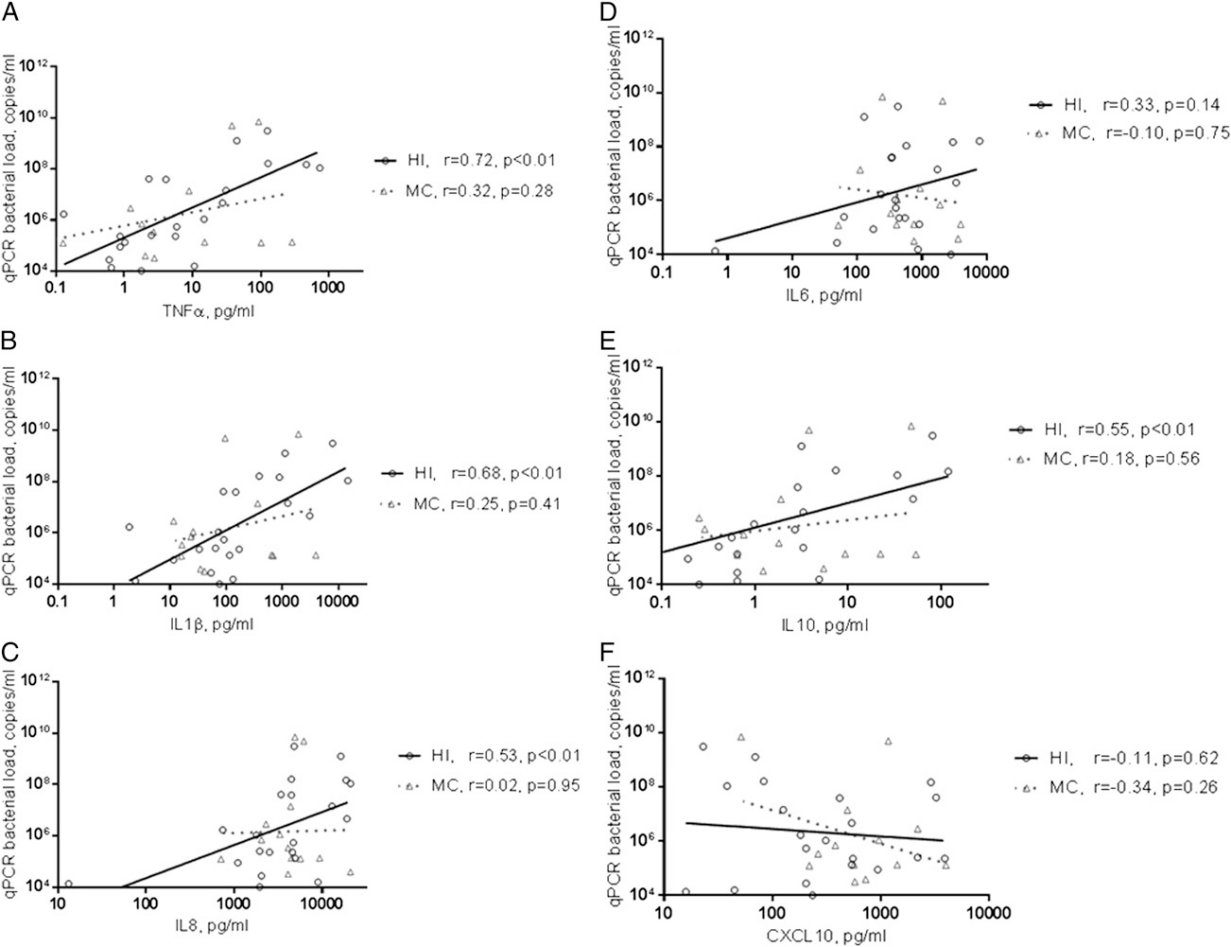
*A-F, Correlations between the sputum mediator concentrations (A) TNF*α*, (B) IL1*β*, (C) IL8, (D) IL6, (E) IL10, and (F) CXCL10 and sputum bacterial load of HI and MC in the subgroup of subjects qPCR positive for a single pathogen only. See Figure 1 and 2 legends for expansion of abbreviations.*

Subjects with pathogen-specific bacterial 16S qPCR PPM had higher levels of IL-1β and TNF-α and lower levels of CCL13 (Table 2, e-Table 3); this was associated with a trend to a dose-response increase in inflammation with increasing numbers of qPCR PPMs (Table 3). There was no correlation of total bacterial 16S qPCR with inflammation. Pathogen-specific 16S qPCR bacterial load in subjects positive for *H influenzae* strongly correlated with levels of IL-1β, IL-8, IL-10, and TNF-α (*r* = 0.64, *P* < .01; *r* = 0.51, *P* < .01; *r* = 0.59, *P* < .01; and *r* = 0.71, *P* < .01, respectively). Pathogen-specific 16S qPCR bacterial load of *M catarrhalis* moderately correlated with IL-10 and TNF-α levels (*r* = 0.31, *P* = .02; and *r* = 0.39, *P* < .01) (Fig 2). Multivariate regression analysis identified qPCR *H influenzae* bacterial load and CFU as independent predictors of sputum TNF-α and IL-1β (*H influenzae*, β = 0.38 and 0.32 for TNF-α and IL-1β, respectively; and CFU, β = 0.36 and 0.31, respectively) (e-Table 4). In subjects with either *H influenzae* or *M catarrhalis* as a single pathogen (n = 21 and n = 13, respectively), only *H influenzae* 16S qPCR bacterial load correlated with IL-1β, IL-10, and TNF-α (Fig 3); and only *H influenzae* qPCR bacterial load correlated with VAS symptoms of cough and sputum purulence and the symptom domain of the SGRQ (e-Fig 1).

**TABLE 2 ]:** Cytokine Levels According to Whether There Was a Pathogen-Specific Bacterial 16S qPCR PPM Detected

Cytokine, pg/mL	qPCR PPM Negative (n = 28)	qPCR PPM Positive (n = 92)	*P* Value
IL-1β	45 (30-68)	142 (93-217)	< .01
IL-5	1.3 (0.7-2.3)	1.0 (0.7-1.4)	.58
IL-6	292 (162-527)	331 (236-464)	.73
IL-8	3,104 (1,940-4,965)	3,707 (2,916-4,712)	.49
IL-10	0.3 (0.2-0.5)	1.8 (1.2-2.7)	< .01
TNF-α	2.2 (1.1-4.2)	7.2 (4.6-11.5)	< .01
TNFRI	834 (573-1,213)	1,222 (1,003-1,489)	.07
CCL2	605 (426-860)	548 (441-682)	.66
CCL3	70 (47-105)	74 (58-94)	.83
CCL4	957 (607-1,510)	989 (737-1,327)	.91
CCL5	3.2 (1.8-5.7)	4.4 (3.5-5.5)	.23
CCL13	47 (31-71)	26 (20-34)	.03
CCL17	33 (20-55)	23 (17-30)	.21
CXCL10	404 (260-627)	252 (188-337)	.12

Data presented as geometric mean (95% CI). TNF = tumor necrosis factor. See Table 1 legend for expansion of other abbreviations.

**TABLE 3 ]:** Parameters of Sputum Inflammation According to Number of qPCR Pathogens Found at Baseline

Parameter	0 qPCR Positive (n = 28)	1 qPCR Positive (n = 38)	≥ 2 qPCR Positive (n = 54)	*P* Value
IL-1β, pg/mL	45 (30-68)	111 (55-222)	169 (100-287)	.01
IL-5, pg/mL	1.3 (0.7-2.3)	1.4 (0.9-2.0)	0.8 (0.5-1.4)	.34
IL-6, pg/mL	292 (162-527)	473 (278-806)	258 (168-395)	.20
IL-8, pg/mL	3,104 (1,940-4,965)	3,578 (2,357-5,434)	3,799 (2,851-5,064)	.77
IL-10, pg/mL	0.3 (0.2-0.5)	1.9 (0.9-3.6)	1.8 (1.0-3.0)	< .01
TNF-α, pg/mL	2.2 (1.1-4.2)	5.4 (2.6-1.4)	8.8 (4.9-15.9)	.02
TNFRI, pg/mL	843 (573-1,213)	1,211 (903-1,623)	1,229 (940-1,608)	.20
CCL2, pg/mL	605 (426-860)	697 (482-1,007)	463 (358-600)	.16
CCL3, pg/mL	70 (47-105)	85 (56-128)	67 (50-90)	.64
CCL4, pg/mL	957 (607-1,509)	1,212 (793-1,851)	858 (574-1,281)	.50
CCL5, pg/mL	3.2 (1.8-5.7)	4.7 (3.3-6.9)	4.2 (3.1-5.5)	.43
CCL13, pg/mL	47 (31-71)	37 (27-51)	21 (14-30)	< .01
CCL17, pg/mL	33 (20-55)	31 (22-44)	18 (12-27)	.07
CXCL10, pg/mL	404 (260-627)	334 (211-529)	207 (142-300)	.07
Log CFU/mL, mean (95% CI)	6.6 (5.4-7.8)	7.3 (5.8-9.1)	7.5 (6.0-9.1)	< .01
Log 16S genome copies/mL, mean (95% CI)	8.2 (6.8-9.4)	8.2 (6.1-9.7)	8.5 (6.8-10.0)	.11
Total cell count, × 10^6^/g	2.5 (1.9-3.7)	3.0 (1.9-4.6)	4.1 (2.8-5.9)	.31
Sputum neutrophils, mean (SEM), %	64 (4)	68 (4)	75 (3)	.06
Sputum neutrophil count, × 10^6^/g	1.6 (0.9-2.8)	1.9 (1.1-3.2)	2.9 (1.9-4.6)	.20
Sputum eosinophils, %	1.6 (1.1-2.2)	1.6 (1.0-2.7)	1.1 (0.8-1.7)	.41

Data presented as geometric mean (95% CI) unless otherwise stated. See Table 1 and 2 legends for expansion of abbreviations.

### qPCR and Inflammation During an Exacerbation

Paired stable and exacerbation sputum was available in 55 subjects (men, n = 43; mean FEV_1_ % predicted, 51%) with an average time of sampling between stable and exacerbation of 49 days. All subjects were treated with oral antibiotic and corticosteroid therapy at the onset of an exacerbation. During an exacerbation, pathogen-specific 16S qPCR for *H influenzae*, *M catarrhalis*, and *S pneumoniae* were detected in 32 (58%), 26 (47%), and 20 (36%) subjects, respectively; the majority of these subjects had the same PPM qPCR at stable state (66% *H influenzae*, 69% *M catarrhalis*, and 55% *S pneumoniae*). In subjects who were pathogen-specific bacterial 16S qPCR PPM positive for *H influenzae* and *M catarrhalis* at exacerbation, the change in cytokine concentration between stable state and exacerbation was most marked in those who were also positive rather than negative in stable state (e-Table 5). The change in pathogen-specific 16S *H influenzae* qPCR bacterial load between stable and exacerbation visits did not correlate with change in cytokines or change in symptoms. The change in pathogen-specific 16S *M catarrhalis* qPCR bacterial load between stable and exacerbation was positively correlated with a change in IL-1β and TNF-α (*r* = 0.37, *P* < .01; *r* = 0.31, *P* = .02, respectively) (e-Fig 2) but was not related to changes in symptoms or health status.

## Discussion

In this study we have shown that the majority of subjects with COPD at stable state had pathogenic bacteria detected by qPCR. The detection of bacteria using qPCR was associated with increased sputum IL-1β, IL-10, and TNF-α and decreased CCL13. In stable state, the strongest relationship between bacterial load, inflammation, and symptoms was observed with pathogen-specific 16S *H influenzae* qPCR, whether in codetection with other bacteria or as a lone pathogen. Furthermore, we determined that *H influenzae* qPCR bacterial load was the only pathogen that was an independent predictor of sputum TNF-α and IL-1β levels, both inflammatory chemokines. In our study, we determined that both total 16S and CFU were elevated in subjects with detectable pathogen-specific 16S but only CFU bacterial load, which suggests live bacteria growth, was independently also associated with increased inflammation. At exacerbation, we have also shown that change of *M catarrhalis*, but not *H influenzae*, bacterial load at exacerbation compared with stable state correlated with change in sputum TNF-α and IL-1β concentrations. Together, these findings suggest there is a complex dynamic relationship in COPD between bacterial load of specific pathogens, airway inflammation, and clinical expression of disease.

Our work represents the largest study to date using qPCR techniques to describe codetection in COPD.^3,27,30^ Prevalence of codetection was not described by Garcha et al,^27^ and, similar to our findings, Curran et al^30^ described, in a small study of 30 subjects with COPD, the presence of codetection using qPCR in 80% of subjects. Consistent with studies using culture-based^15,16^ and culture-independent^27^ techniques, we report here that *H influenzae* was the most commonly identified organism by qPCR. The presence of any detectable PPM was associated with increased airway inflammation and increased proinflammatory cytokines. Our data suggest that sputum TNF-α and IL-1β are more closely related to bacterial load, particularly with *H influenzae*, which was found to be an independent predictor of sputum TNF-α and IL-1β levels and associated with increased symptoms. Although this association does not confirm causality, there are several biologically plausible reasons that *H influenzae* may be of most significance. First, up-regulation of the MRLP3 inflammasome occurs during nontypeable *H influenzae*-induced inflammation leading to secretion of IL-1β^31^; second, the outer membrane protein P6 of nontypeable *H influenzae* has been found to induce the stimulation of TNF-α and IL-10 from human alveolar macrophages.^32,33^ Furthermore, alveolar macrophages have the greatest reduction of complement-independent phagocytosis of nontypeable *H influenzae*.^34^ However, it must be noted that qPCR techniques measure both live and dead bacteria. Our observation that CFU bacterial load and *H influenzae* 16S pathogen-specific bacterial load were closely associated with sputum levels of TNF-α and IL-1β may suggest that live *H influenzae* is driving this inflammation, but further studies to investigate this are warranted.

We also observed that CCL13, which is increased in eosinophilic airway disease,^35^ was reduced in subjects with pathogen-specific 16S qPCR PPM detection, irrespective of pathogen. Although we could not show that there is increased neutrophilic inflammation, with elevated IL8 or sputum neutrophils percent as demonstrated in previous studies,^11,36^ there was a trend to increase in total cell counts and absolute sputum neutrophil counts as codetection of pathogen increased and a positive correlation of IL-8 with bacterial load, further suggesting a differential airway pattern of inflammation with qPCR PPM detection. IL-10, an antiinflammatory cytokine, was also found to be increased in subjects with pathogen-specific 16S qPCR PPM and correlated with *H influenzae* bacterial load. Specifically, the outer membrane protein P6 of *H influenzae* has been shown to be a potent macrophage inducer of inflammation,^33^ and chronic upregulation and stimulation of macrophages may contribute to further to the pathogenesis of COPD infections.^37^

In contrast to earlier reports, we were unable to detect significant differences in exacerbation frequency, smoking, or inhaled corticosteroid dose between colonized and noncolonized subjects.^27^ However, we have demonstrated a positive association between symptoms and *H influenzae* bacterial load. We report here that the relationship between bacterial load and symptoms is pathogen specific and again supports a central role for sputum *H influenzae* bacterial load in chronic persistent disease. Whether therapy specifically targeted at reducing *H influenzae* bacterial load during stable state to reduce inflammatory activity in COPD is clinically beneficial is currently unknown and warrants further study.

Previous studies have demonstrated that airway inflammation increases during exacerbation episodes^3,12^ and that the presence of PPM during exacerbations is associated with increased IL-8, IL-1β, and TNF-α.^3,38,39^ However, limited data examining relationships between specific pathogens and inflammation during exacerbations exist. To our knowledge, this is the first study to explore relationships between dynamic changes in pathogen-specific bacterial load and inflammation at exacerbation. We found that change in inflammatory mediators at exacerbation compared with stable state was greatest in subjects who were pathogen-specific bacterial 16S qPCR PPM positive for either *H influenzae* or *M catarrhalis* at stable state. This suggests that there is activation of inflammation in stable state and colonized subjects with COPD, which is then further exaggerated at exacerbation. At stable state, *H influenzae* is closely related to airway inflammation and clinical outcomes; however, at exacerbation, changes in inflammation are more closely related to changes (including increases and decreases) of pathogen-specific bacterial 16S qPCR *M catarrhalis* bacterial loads. The role of this pathogen during exacerbations of COPD needs to be investigated further.

One potential limitation of this study was the use of spontaneous sputum to determine sputum inflammatory mediators and pathogen detection. Sputum collection predominately arises from the larger airways; thus, our results cannot infer causality of detectable pathogens and increased inflammation in the pathogenicity of COPD, a predominately small airways disease. However, only samples with a squamous cell contamination < 5% were used, and inflammatory mediators measured from induced and spontaneous samples have not been shown to be different,^24^ suggesting that the strong associations found between airway bacterial load and sputum inflammatory mediator concentrations were not clinically significant. During this study, we also selected cutoffs to determine the presence or absence of pathogen-specific 16S qPCR. Although this is an arbitrary cutoff, we acknowledge that this is in part because of a paucity of the literature, which may be missing or overdetecting pathogens, but we have used cutoffs derived from previous studies using qPCR platforms in patients with COPD.^27^ We have defined the presence of pathogen in sputum samples as detection and not colonization in this study, as the stable measurements were performed only at one time point, and we cannot comment on whether there is transient detection of pathogens using qPCR methods or persistence; it is clear that more work is needed to interrogate the stability and repeatability of pathogen-specific 16S qPCR in patients with COPD. A further limitation is that the presence of *P aeruginosa* was not assessed using qPCR techniques in this study. Although previous culture studies have suggested airway inflammation is higher in subjects in whom *P aeruginosa* is detected,^14^ its prevalence in other COPD cohorts has been low.^27^ Moreover, within our cohort, only five of 120 patients had grown *P aeruginosa* on sputum cultures within the preceding 12 months, and hence its exclusion from our analysis is unlikely to have significantly influenced our results. In our study we chose primers targeting specific pathogens in the respiratory tract, as they have previously been deemed clinically significant. However, these techniques cannot reflect the total microbiologic diversity within the airway, and newer techniques that can characterize the entire microbiome need to be studied further.^40‐42^ Finally, measuring sputum mediators can be fraught with difficulty, with variability in detection^43‐45^; however, we have only analyzed mediators that were known to be detectable using a sputum-processing method that we have previously validated in patients with airway disease.^25^

## Conclusions

In summary, using qPCR we found that in patients with COPD, sputum pathogens are frequently detected. *H influenzae* was associated with increased airway inflammation and symptoms in a dose-response relationship with sputum TNF-α and IL-1β. *M catarrhalis* was more closely related to dynamic changes observed at exacerbation. The mechanisms by which *H influenzae* and *M catarrhalis* are related to airway inflammation in COPD warrant further investigation.

## Supplementary Material

Online Supplement

## ABBREVIATIONS

CFU: colony-forming units
PPM: potentially pathogenic microorganism
qPCR: quantitative polymerase chain reaction
SGRQ: St. George’s Respiratory Questionnaire
TNF: tumor necrosis factor
VAS: visual analog scale
