# Supplementary material for: Association Between Pathogens Detected Using Quantitative Polymerase Chain Reaction With Airway Inflammation in COPD at Stable State and Exacerbations
Source: Chest. 2014 Aug 7;147(1):46–55. doi: 10.1378/chest.14-0764 (PMC4285081; doi:10.1378/chest.14-0764)
Supplement: Supplementary file 1 [file chest_147_1_46_ds01.pdf]

CHEST Online Supplement

Association Between Pathogens Detected Using Quantitative Polymerase Chain Reaction With Airway Inflammation in COPD at Stable State and Exacerbations

Bethan L. Barker, BMBS; Koirobi Haldar, MSc; Hemu Patel, BSc; Ian D. Pavord, MD; Michael R. Barer, PhD; Christopher E. Brightling, PhD, FCCP; and Mona Bafadhel, PhD

CHEST 2015; 147(1):46–55

e-Table 1: Target genes and primers for each of the qPCR reaction assays used

Target organism	Target gene	Primers	Sequence
Total bacteria	16S rDNA	338F<br>515R	5'ACTCCTACGGGNGGCNGCA3'<br>5'GTATTACCGCNNCTGCTGGCAC3'
S. pneumoniae	Pneumolysin	*S. pneumoniae* F<br>*S. pneumoniae* R<br>*S. pneumoniae* probe	5'AGCGATAGCTTTCTCCAAGTGG3'<br>5'CTTAGCCAACAAATCGTTTACCG3'<br>5'Cy5-ACCCCAGCAATTCAAGTGTTCGCG-BHQ2-3'
H. influenzae	Outer membrane protein P6	P6F<br>P6R	5'TTGGCGGWTACTCCTGTTGCT3'<br>5'TGCAGGTTTTTCTTCACCGT3'
S. aureus	Thermonuclease	*S. aureus* (nuc) F<br>*S. aureus* (nuc) R	5'GCGATTGATGGTGATACGGTT3'<br>5'AGCCAAGCCTTGACGAACTAAAGC3'
M. catarrhalis	Outer membrane protein CopB	*M. catarrhalis* F<br>*M. catarrhalis* R<br>*M. catarrhalis* probe	5'GTGAGTGCCGCTTTACAACC3'<br>5'TGTATCGCCTGCCAAGACAA3'<br>5'JOE-TGCTTTTGCAGCTGTTAGCCAGCCTAA-TAMRA3'

Online supplements are not copyedited prior to posting.

© 2015 AMERICAN COLLEGE OF CHEST PHYSICIANS. Reproduction of this article is prohibited without written permission from the American College of Chest Physicians. See online for more details. **DOI:** 10.1378/chest.14-0764

CHEST Online Supplement

e-Table 2: Lower limit of detection for measured cytokines using MSD®

pg/ml	Lower limit of detection
IL1β	0.13
IL5	0.13
IL6	0.13
IL8	0.13
IL10	1.15
TNFα	1.32
TNFRI	16.00
CCL2	3.20
CCL3	7.47
CCL4	3.20
CCL5	2.18
CCL13	5.76
CCL17	0.47
CXCL10	3.20

Online supplements are not copyedited prior to posting.

© 2015 AMERICAN COLLEGE OF CHEST PHYSICIANS. Reproduction of this article is prohibited without written permission from the American College of Chest Physicians. See online for more details. **DOI:** 10.1378/chest.14-0764

CHEST™ Online Supplement

e-Table 3: Sputum inflammatory mediators according to whether or not individual pathogens were detected by qPCR

	H. influenzae –ve (n=49)	*H. influenzae* +ve (n=71)	p-value	*M. catarrhalis* -ve (n=62)	M. catarrhalis +ve (n=58)	p-value	*S. pneumoniae* –ve (n=86)	*S. pneumoniae* +ve (n=34)	p-value
IL1β, pg/ml	56 (36-86)	172 (107-277)	**<0.01**	71 (46-110)	170 (100-289)	**0.01**	111 (73-169)	102 (55-189)	0.84
IL5, pg/ml	1.3 (0.8-2.0)	1.0 (0.6-1.4)	0.32	1.2 (0.9-1.7)	0.9 (0.6-1.5)	0.34	1.3 (1.0-1.8)	0.7 (0.3-1.2)	**0.03**
IL6, pg/ml	398 (262-604)	279 (187-415)	0.24	310 (205-468)	335 (221-508)	0.79	371 (261-527)	225 (135-376)	0.13
IL8, pg/ml	3455 (2524-4731)	3627 (2714-4849)	0.83	3151 (2278-4358)	4049 (2071-5319)	0.25	3538 (2720-4603)	3601 (2514-5161)	0.94
IL10, pg/ml	0.7 (0.4-1.2)	1.8 (1.1-2.9)	**0.01**	0.6 (0.4-1.1)	2.3 (1.4-3.8)	**<0.01**	1.1 (0.7-1.8)	1.4 (0.7-2.9)	0.68
TNFα, pg/ml	2.8 (1.6-4.9)	8.7 (5.2-14.6)	**<0.01**	3.2 (1.9-5.3)	9.8 (5.6-17.3)	**<0.01**	5.3 (3.3-8.3)	6.1 (2.7-13.6)	0.74
TNFRI, pg/ml	962 (742-1248)	1240 (978-1571)	0.17	933 (735-1184)	1356 (1051-1749)	**0.04**	1161 (942-1430)	1015 (727-1418)	0.51
CCL2, pg/ml	634 (482-833)	516 (402-663)	0.29	634 (483-832)	493 (385-632)	0.19	631 (507-786)	417 (299-582)	0.05
CCL3, pg/ml	72 (50-102)	74 (57-96)	0.90	71 (54-92)	76 (55-105)	0.75	78 (60-102)	61 (45-85)	0.31
CCL4, pg/ml	1070 (744-1541)	925 (660-1296)	0.57	910 (679-1219)	1065 (708-1603)	0.54	1074 (780-1479)	782 (562-1089)	0.26
CCL5, pg/ml	3.3 (2.2-5.0)	4.7 (3.7-6.0)	0.13	3.8 (2.8-5.3)	4.4 (3.3-5.8)	0.57	4.3 (3.3-5.6)	3.6 (2.4-5.4)	0.52
CCL13, pg/ml	38 (29-51)	25 (19-35)	0.08	40 (31-53)	22 (16-31)	0.14	33 (25-43)	24 (17-34)	0.19
CCL17, pg/ml	32 (23-45)	21 (15-29)	0.10	30 (22-40)	20 (13-31)	0.14	29 (21-39)	17 (11-27)	0.07
CXCL10, pg/ml	390 (278-546)	225 (150-316)	**0.03**	296 (213-410)	267 (183-389)	0.69	325 (240-440)	196 (132-292)	0.07
Log colony forming units/ml*	6.7 (5.6-8.1)	7.6 (5.9-9.1)	**<0.01**	7.1 (5.7-9.0)	7.4 (5.9-9.1)	0.05	7.1 (5.8-9.1)	7.5 (5.8-9.1)	0.10
Log 16S genome copies/ml	8.1 (6.1-9.7)	8.5 (6.9-9.8)	**0.05**	8.4 (7.1-9.6)	8.3 (6.2-9.9)	0.64	8.2 (6.5-9.6)	8.8 (6.9-10.1)	**<0.01**
Total cell count, $x10^6$/g	2.6 (1.7-3.9)	3.9 (2.9-5.4)	0.11	2.8 (2.0-4.0)	3.9 (2.7-5.6)	0.20	3.4 (2.5-4.5)	3.2 (2.0-4.9)	0.83
Sputum neutrophils %~	66 (3)	73 (3)	0.08	66 (3)	75 (3)	**0.02**	69 (2)	72 (4)	0.63
Sputum eosinophils %	1.3 (0.9-1.8)	1.4 (1.0-2.1)	0.67	1.8 (1.2-2.6)	1.0 (0.7-1.4)	0.03	1.5 (1.1-2.0)	1.2 (0.7-2.0)	0.47

Data presented as geometric mean (95% CI) unless otherwise stated; *mean (95% CI); ~mean (SEM)

Online supplements are not copyedited prior to posting.

© 2015 AMERICAN COLLEGE OF CHEST PHYSICIANS. Reproduction of this article is prohibited without written permission from the American College of Chest Physicians. See online for more details. **DOI:** 10.1378/chest.14-0764

CHEST Online Supplement

e-Table 4

(a) Multivariate regression analysis to determine independent relationships with sputum TNFα in cohort of n=120, R^2=0.62

Variable	Beta coefficient	p-value
FEV_1 % predicted	0.04	0.69
Exacerbation frequency	0.03	0.76
H. influenzae bacterial load	0.37	**<0.01**
M. catarrhalis bacterial load	0.08	0.48
Colony forming units	0.36	**<0.01**
Sputum total cell count	0.18	0.16
Sputum neutrophil %	0.07	0.48

(b) Multivariate regression analysis to determine independent relationships with sputum IL1β in cohort of n=120, R^2=0.51

Variable	Beta coefficient	p-value
FEV_1 % predicted	0.12	0.25
Exacerbation frequency	0.02	0.87
H. influenzae bacterial load	0.32	**0.02**
M. catarrhalis bacterial load	-0.07	0.54
Colony forming units	0.31	**0.02**
Sputum total cell count	0.23	0.09
Sputum neutrophil %	0.21	0.10

Online supplements are not copyedited prior to posting.

© 2015 AMERICAN COLLEGE OF CHEST PHYSICIANS. Reproduction of this article is prohibited without written permission from the American College of Chest Physicians. See online for more details. **DOI:** 10.1378/chest.14-0764

e-Table 5: Sputum mediators according to change in (a) *H. influenzae*, (b) *M. catarrhalis* and (c) *S. pneumoniae* qPCR status between stable and exacerbation visits

(a)

	HI positive/positive (n=21)			HI negative/positive (n=11)		
	Stable	Exacerbation	p-value	Stable	Exacerbation	p-value
IL1β, pg/ml	190 (132-274)	615 (427-886)	**<0.01**	20 (11-36)	34 (11-111)	0.25
IL5, pg/ml	0.7 (0.5-1.0)	0.6 (0.4-0.9)	**<0.01**	1.0 (0.6-1.6)	1.0 (0.4-2.3)	0.95
IL6, pg/ml	32 (224-465)	754 (523-1086)	**<0.01**	458 (277-757)	600 (177-2029)	0.69
IL8, pg/ml	6595 (4578-9503)	9518 (6606-13713)	0.13	2742 (1794-4190)	1854 (751-4575)	0.33
IL10, pg/ml	3.0 (2.1-4.3)	8.7 (6.1-12.6)	**0.04**	0.2 (0.1-0.6)	1.1 (0.4-3.1)	**0.03**
TNFα, pg/ml	21.2 (14.7-30.5)	62.6 (43.5-90.2)	**<0.01**	1.4 (0.6-3.0)	6.3 (1.5-27.1)	**0.03**
TNFRI, pg/ml	1526 (1059-2199)	2970 (2061-4279)	0.20	753 (554-1025)	808 (446-1464)	0.83
CCL2, pg/ml	487 (338-702)	376 (261-541)	0.09	524 (290-945)	570 (318-1024)	0.80
CCL3, pg/ml	88 (61-126)	125 (86-179)	0.41	37 (26-52)	53 (22-132)	0.31
CCL4, pg/ml	1045 (725-1506)	1736 (1205-2501)	0.09	521 (274-990)	876 (290-2652)	0.41
CCL5, pg/ml	4.9 (3.4-7.1)	8.1 (5.6-11.6)	0.16	2.1 (1.4-3.2)	3.5 (1.6-7.4)	0.25
CCL13, pg/ml	13.8 (9.6-19.9)	10.1 (7.0-14.5)	**0.04**	33 (26-42)	18 (10-34)	0.06
CCL17, pg/ml	12.8 (8.9-18.5)	5.9 (4.1-8.6)	**0.01**	21 (14-30)	21 (11-43)	0.91
CXCL10, pg/ml	209 (145-302)	283 (196-407)	**0.03**	245 (117-513)	521 (158-1722)	0.31
Total cell count, x10^6/g	5.8 (4.0-8.3)	12.4 (8.6-17.8)	**0.02**	1.7 (0.9-3.4)	2.0 (0.9-4.6)	0.79
Sputum neutrophils %¬	76 (4)	80 (5)	0.12	54 (7)	60 (8)	0.51
Sputum eosinophils %	1.4 (1.0-2.1)	0.6 (0.4-0.8)	**<0.01**	1.8 (0.9-3.3)	2.5 (1.0-6.3)	0.53

Data expressed as geometric mean (95% CI); ¬ mean (SEM)

Online supplements are not copyedited prior to posting.

© 2015 AMERICAN COLLEGE OF CHEST PHYSICIANS. Reproduction of this article is prohibited without written permission from the American College of Chest Physicians. See online for more details. **DOI:** 10.1378/chest.14-0764

(b)

	MC positive/positive (n=18)			MC negative/positive (n=8)		
	Stable	Exacerbation	p-value	Stable	Exacerbation	p-value
IL1β, pg/ml	138 (53-356)	507 (217-1187)	**0.02**	55 (25-123)	162 (39-675)	0.18
IL5, pg/ml	0.7 (0.4-1.3)	0.6 (0.3-1.2)	0.36	2.8 (1.1-6.7)	1.9 (0.8-4.7)	0.21
IL6, pg/ml	655 (360-1191)	867 (473-1588)	0.34	536 (3.5-943)	1023 (506-2069)	0.28
IL8, pg/ml	7640 (4711-12389)	9762 (6891-13829)	0.32	5563 (2848-10863)	5050 (2449-10412)	0.82
IL10, pg/ml	2.9 (1.0-8.3)	7.1 (2.6-19.5)	0.15	0.9 (0.3-2.9)	2.4 (1.0-5.4)	0.23
TNFα, pg/ml	15.5 (5.8-41.3)	49.4 (19.8-123.4)	**0.03**	8.4 (2.5-28.3)	28.0 (5.4-146.4)	0.20
TNFRI, pg/ml	1711 (1136-2578)	2632 (1804-3840)	0.12	1153 (717-1853)	1527 (836-2787)	0.49
CCL2, pg/ml	433 (289-648)	552 (312-976)	0.32	951 (489-1850)	562 (318-995)	0.18
CCL3, pg/ml	68 (47-100)	120 (65-220)	0.15	104 (55-197)	102 (57-180)	0.96
CCL4, pg/ml	1049 (720-1529)	1666 (969-2864)	0.13	1484 (991-2222)	1591 (840-3013)	0.85
CCL5, pg/ml	3.7 (2.7-5.0)	7.7 (4.2-14.2)	**0.03**	5.2 (3.0-9.0)	5.3 (2.8-10.2)	0.96
CCL13, pg/ml	23.3 (13.7-39.5)	14.0 (8.1-24.3)	0.15	37.3 (16.5-84.4)	21.2 (11.1-40.6)	0.22
CCL17, pg/ml	17.0 (8.2-35.4)	9.5 (5.0-18.1)	0.13	26.2 (12.5-55.1)	11.3 (3.9-32.9)	0.03
CXCL10, pg/ml	224 (109-459)	303 (120-764)	0.47	519 (148-1817)	398 (160-988)	0.75
Sputum total cell count, $x10^6$/g	3.3 (1.4-7.9)	5.4 (2.8-10.4)	0.21	3.4 (2.2-5.2)	7.2 (4.5-11.5)	0.10
Sputum neutrophils %¬	74 (5)	71 (7)	0.77	69 (7)	72 (6)	0.65
Sputum eosinophils %	1.1 (0.5-2.1)	0.9 (0.4-2.4)	0.72	2.8 (1.2-6.6)	1.1 (0.3-5.0)	0.31

Data expressed as geometric mean (95% CI); ¬ mean (SEM)

Online supplements are not copyedited prior to posting.

© 2015 AMERICAN COLLEGE OF CHEST PHYSICIANS. Reproduction of this article is prohibited without written permission from the American College of Chest Physicians. See online for more details. **DOI:** 10.1378/chest.14-0764

(c)

	SP positive/positive (n=11)			SP negative/positive (n=9)		
	Stable	Exacerbation	p-value	Stable	Exacerbation	p-value
IL1β, pg/ml	211 (68-658)	521 (205-1326)	0.12	79 (32-199)	274 (63-1193)	0.05
IL5, pg/ml	0.6 (0.3-1.2)	0.6 (0.3-1.1)	0.97	1.2 (0.6-2.4)	1.8 (0.7-5.0)	0.42
IL6, pg/ml	165 (71-380)	487 (252-940)	**0.02**	844 (416-1713)	1129 (454-2811)	0.59
IL8, pg/ml	5745 (2520-13101)	9425 (6419-13839)	0.21	5022 (2610-9665)	10091 (6151-16554)	**<0.01**
IL10, pg/ml	2.6 (0.7-9.3)	7.9 (2.2-28.1)	0.08	1.9 (0.5-8.4)	5.6 (1.2-27.1)	0.32
TNFα, pg/ml	25.1 (6.2-101.1)	76.1 (25.3-228.7)	0.05	6.5 (2.0-21.6)	23.5 (4.6-119.9)	0.14
TNFRI, pg/ml	1415 (680-2945)	2441 (1476-4035)	0.22	1180 (653-2133)	2929 (1542-5564)	**<0.01**
CCL2, pg/ml	368 (239-569)	392 (241-639)	0.79	353 (183-683)	802 (369-1744)	**0.03**
CCL3, pg/ml	75 (46-120)	86 (49-152)	0.59	94 (41-216)	168 (63-453)	0.27
CCL4, pg/ml	704 (456-1089)	1112 (852-1453)	0.06	1028 (575-1840)	2343 (1044-5258)	0.06
CCL5, pg/ml	4.0 (2.6-6.0)	8.1 (3.7-17.8)	0.06	4.7 (2.0-11.2)	10.7 (4.1-28.0)	0.10
CCL13, pg/ml	12.8 (7.4-22.1)	6.9 (4.4-10.9)	0.19	37.1 (20.5-67.0)	29.8 (21.4-41.5)	0.54
CCL17, pg/ml	9.9 (4.1-23.5)	3.5 (1.9-6.5)	0.09	22.3 (7.7-64.5)	34.4 (17.9-66.2)	0.55
CXCL10, pg/ml	129 (63-265)	236 (69-810)	0.21	480 (205-1127)	619 (168-2277)	0.69
Sputum total cell count, $x10^6$/g	3.1 (1.2-8.1)	6.4 (2.7-14.9)	0.24	3.4 (1.3-9.2)	3.6 (1.5-8.9)	0.90
Sputum neutrophils %¬	74 (8)	83 (5)	0.23	65 (7)	67 (8)	0.90
Sputum eosinophils %	0.8 (0.3-2.0)	0.5 (0.2-1.2)	0.51	1.4 (0.6-3.3)	1.4 (0.5-4.1)	0.95

Data expressed as geometric mean (95% CI); ¬ mean (SEM)

Online supplements are not copyedited prior to posting.

© 2015 AMERICAN COLLEGE OF CHEST PHYSICIANS. Reproduction of this article is prohibited without written permission from the American College of Chest Physicians. See online for more details. **DOI:** 10.1378/chest.14-0764

CHEST Online Supplement

e-Figure 1. Correlations between visual analogue scores (VAS) or St George's questionnaire symptom score and sputum bacterial load of *H. influenzae* (HI), *M. catarrhalis* (MC) and *S. pneumoniae* (SP)

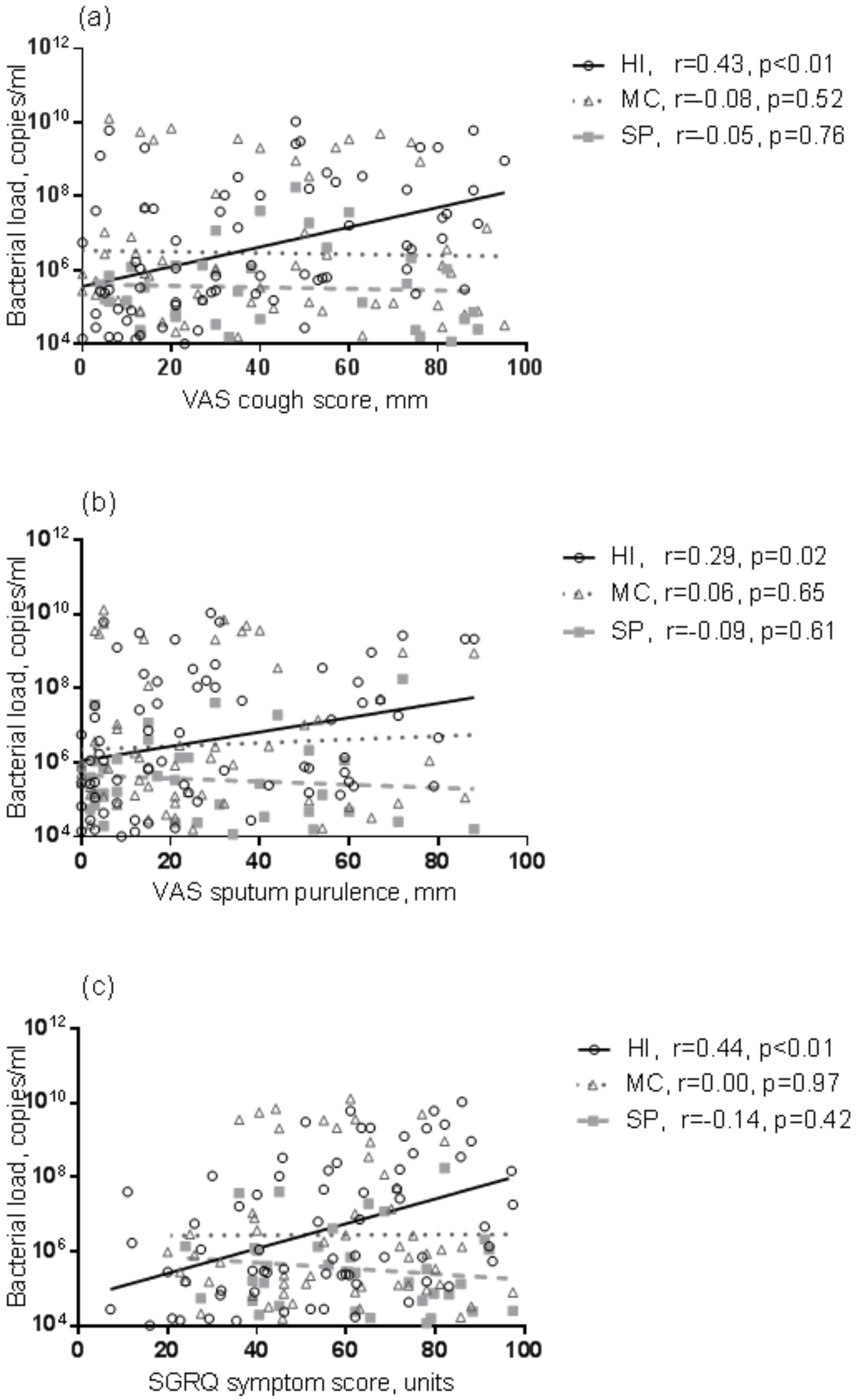

Online supplements are not copyedited prior to posting.

© 2015 AMERICAN COLLEGE OF CHEST PHYSICIANS. Reproduction of this article is prohibited without written permission from the American College of Chest Physicians. See online for more details. **DOI:** 10.1378/chest.14-0764

e-Figure 2. Correlations between log change (D) of the sputum mediator concentrations for (a) IL1β and (b) TNFα and log change (D) of qPCR *M. catarrhalis* load between stable and exacerbation visits (n=55)

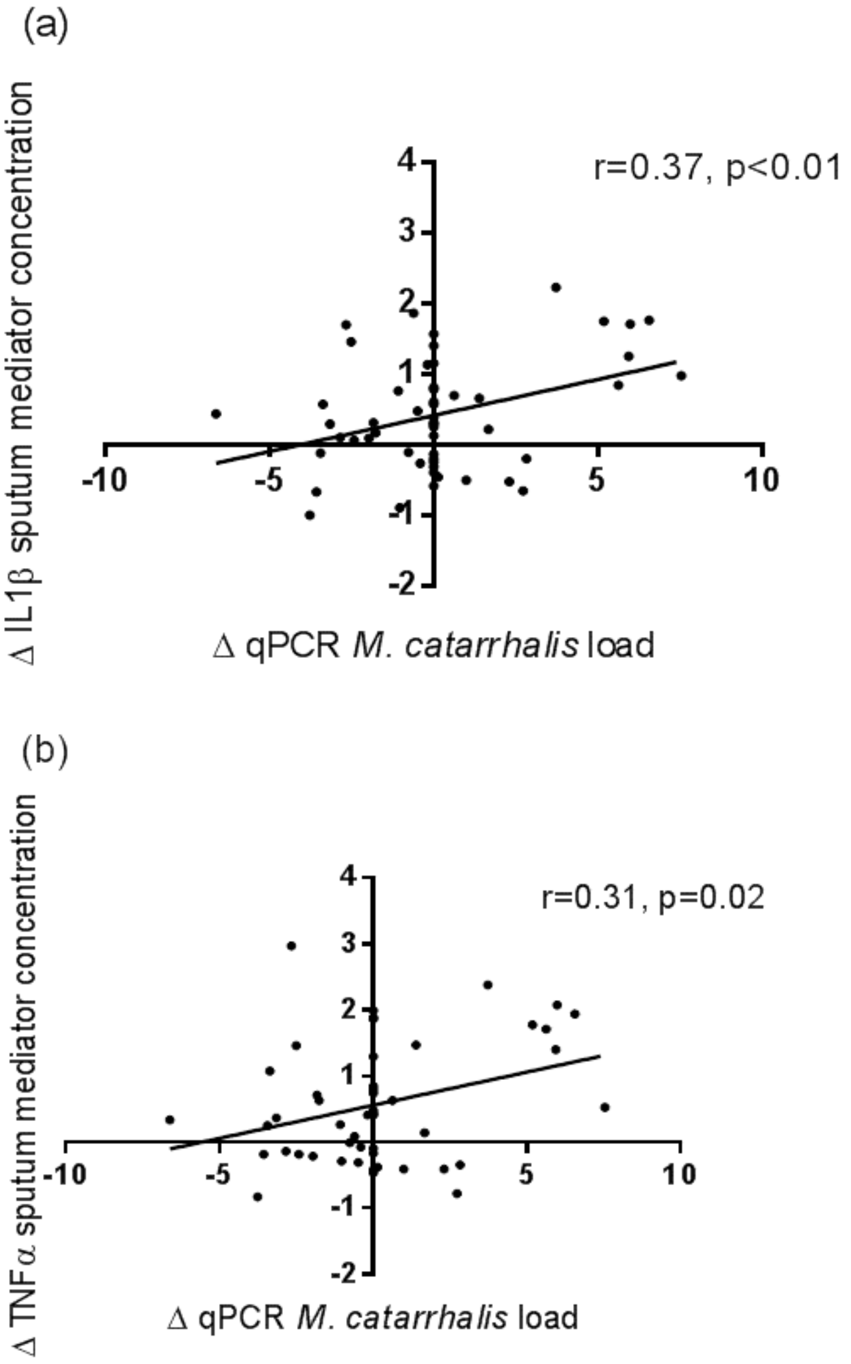

Online supplements are not copyedited prior to posting.

© 2015 AMERICAN COLLEGE OF CHEST PHYSICIANS. Reproduction of this article is prohibited without written permission from the American College of Chest Physicians. See online for more details. **DOI:** 10.1378/chest.14-0764
